# Enhancing the Design of Experiments on the Fatigue Life Characterisation of Fibre-Reinforced Plastics by Incorporating Artificial Neural Networks

**DOI:** 10.3390/ma17030729

**Published:** 2024-02-03

**Authors:** Christian Witzgall, Moh’d Sami Ashhab, Sandro Wartzack

**Affiliations:** 1 Engineering Design, Friedrich-Alexander-Universität Erlangen-Nürnberg, 91058 Erlangen, Germany; wartzack@mfk.fau.de; 2 Mechanical Engineering Department, Al Hussein Technical University, Amman 11831, Jordan; 3 Mechanical Engineering Department, Hashemite University, Zarqa 13115, Jordan

**Keywords:** fatigue life, artificial neural networks, design of experiments, increased efficiency, short-fibre-reinforced thermoplastics, composites

## Abstract

Fatigue life testing is a complex and costly matter, especially in the case of fibre-reinforced thermoplastics, where other parameters in addition to force alone must be taken into account. The number of tests required therefore increases significantly, especially if the influence of different fibre orientations is to be taken into account. It is therefore important to gain the greatest possible amount of knowledge from the limited number of available tests. In order to achieve this, this study aims to utilise adaptive sampling, which is used in numerous areas of computational engineering, for the design of experiments on fatigue life testing. Artificial neural networks (ANNs) are therefore trained on data for the short-fibre-reinforced material PBT GF30, and their areas of greatest model uncertainty are queried. This was undertaken with ANNs from various numbers of hidden layers, which were analysed for their performance. The ideal case turned out to be four hidden layers, for which a squared error as small as 1 × 10^−3^ was recorded. Locally resolved, the ANN was used to identify the region of greatest uncertainty for samples of vertical orientation and small numbers of cycles. With information such as this, additional data can be obtained in such uncertain regions in order to improve the model prediction—almost halving the recorded error to only 0.55 × 10^−3^. In this way, a model of comparable value can be found with less experimental effort, or a model of better quality can be set up with the same experimental effort.

## 1. Introduction

### 1.1. Motivation

Fibre-reinforced plastics have outstanding mechanical properties combined with a low density [1]. They are therefore important materials in lightweight design and are used in a wide range of applications, from the automotive industry [2] to aviation [3] or from plant design to the transportation sector [4]. The range of materials belonging to this material class extends from short-fibre- or long-fibre-reinforced thermoplastics produced by injection moulding for large-scale production [5,6] to hand-laminated individual parts made from continuous fibre-reinforced laminates [7]. However, what all of them have in common is that the material properties are significantly influenced by the manufacturing process. This means that material data cannot be taken at random from data sheets, but must often be characterised for the specific application [8].

One area in which knowledge of material behaviour is of great importance is fatigue: it is important to be able to predict the service life of a component under the application loads [9]. However, the determination of material data in the field of fatigue is often very time-consuming and cost-intensive due to long test durations [10,11]. S-N curves must be determined, which, unlike for metallic materials, depend not only on the load but also on other parameters, such as the fibre orientation [12,13]. This significantly increases the number of tests required. This article therefore aims to show how the use of artificial neural networks can improve test planning. The aim is either to achieve a prediction of the same quality with fewer tests or a prediction of better quality with the same number of tests.

### 1.2. State of the Art in Fatigue Assessment of Fibre-Reinforced Plastics

#### 1.2.1. Determination of S-N Curves: Horizon Method and Pearl–String Method

**Figure 1:**
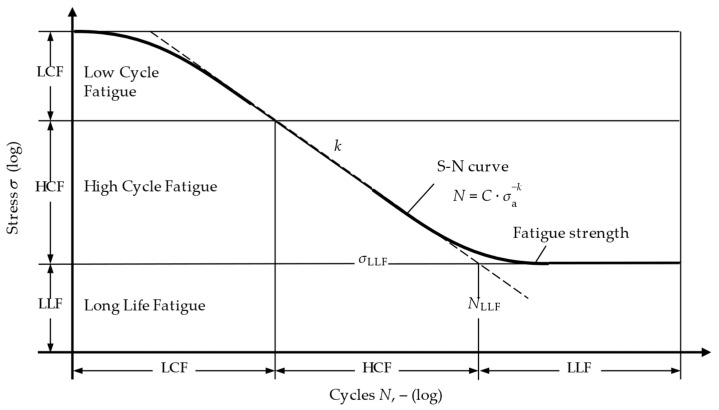
Typical S-N curve with the regions of low-cycle (LCF), high-cycle (HCF) and long-life fatigue (LLF).

The question of which service life can be expected at which load level must be answered using S-N curves. They represent a relationship between the existing stress (S) and the endured cycles (N). The tests are generally carried out by subjecting test specimens to cyclic loads of constant amplitude until they fail. An S-N curve is formed from the available test results, as shown in Figure 1 [14]. It is categorised into regions of low-cycle (LCF), high-cycle (HCF) and long-life (LLF) fatigue. The exact definition of the limits of these regions differs depending on the material and the source used [15]—but should be disregarded at this point. It is more important to understand that an S-N curve, such as the one shown, only represents the exact sample and test condition with which it was recorded. A change in parameters such as fibre the orientation or stress ratio makes it necessary to measure a separate S-N curve [16].

**Figure 2:**
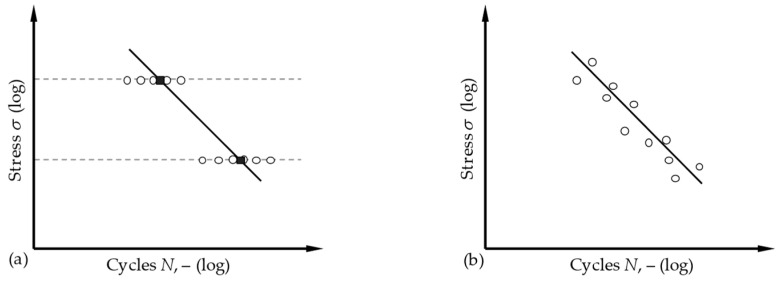
Fatigue testing methods in comparison: horizon method (**a**) vs. pearl–string method (**b**) [17].

The experimental characterisation of such an S-N curve is standardised in accordance with DIN 50100 [17,18]. It essentially proposes two methods, the horizon method and the pearl–string method, which are shown in Figure 2. In the horizon method, two load horizons are selected, on which several repeat tests are carried out. This offers the advantage that the scatter of the number of cycles can be analysed for each selected load level. In order to determine the S-N curve as accurately as possible, it is important that the load levels are selected in such a way that their results cover the range of the HCF as far as possible. However, in order to achieve this, prior knowledge of the material behaviour is always required. As that knowledge is not always known, it is a clear disadvantage of the horizon method.

This is exactly where the pearl–string method comes in and aims to make it possible to analyse a previously unknown material for which the load levels of the limits of the HCF range cannot be estimated. It starts with a fairly arbitrary load level; the subsequent load levels are determined on the basis of the results of the previous tests: if the service life achieved is low, the load is reduced in order to achieve a higher number of cycles in the next test and vice versa. In this way, the course of the S-N curve is scanned iteratively, i.e., the test points are strung together like a string of pearls.

A clear advantage of the pearl–string method is that no prior knowledge of the material to be tested is required, and yet an S-N curve can be reliably determined. However, it can only be assumed, for example, that the scatter of the test results is constant over the entire load range. Furthermore, it must be noted that the standard for the pearl–string method does not provide any information on the extent to which the load should be varied; it only provides the general information: increase or decrease the load.

#### 1.2.2. Consideration of Fibre Orientation

The methods described only provide for the load as a variable parameter in the experiment. However, the load is only one parameter among several, at least in the case of testing fibre-reinforced plastics. As the least considered requirement, the fibre orientation of a sample relative to the force direction of the testing machine must be taken into account. Its influence is the most relevant. Usually, dog-bone-shaped moulds are used as the specimen geometry, but rectangular plates without a waist are also tested less frequently [19].

The orientations 0°, 45° and 90° relative to the test force are frequently tested; more rarely, only the parallel and vertical orientations are tested [20]. Other intermediate angles, such as 30° or 60°, are added less frequently [13]. The samples used are usually taken from injection-moulded plates by machining or water-jet cutting [21]. In addition to the mere orientation, the position within the plate is also changed in places [22].

Of course, the number of realistically feasible different fibre orientations in the samples is limited. Modelling approaches have therefore been developed that allow the interpolation of any orientation angle. One example is the concept of the master S-N curve according to Bernasconi et al. [13], in which no absolute values are used for the stress level, but the stress is considered in relation to the static breaking load.

Attempts have mainly so far been made to make the best possible use of the available test results by means of downstream modelling procedures. Approaches to take better account of the requirements of subsequent modelling as early as the test planning stage have not yet been available to a sufficient extent if other parameters need to be varied instead of the test load alone.

#### 1.2.3. Further Influences on Fatigue Strength

If other influences, such as the stress ratio or temperature, are of interest, these would also have to be varied in a suitable manner, which is not provided for in the standardisation. In order to be able to investigate fatigue strength under multi-axial loading, Moosbrugger, Monte et al. [23,24] designed a specially injection-moulded, tapered tube specimen that can be tested simultaneously under axial force and torsion [24]. Marco et al. used a perforated, injection-moulded sample to determine the energy dissipation [25]. Furthermore, the ratio of upper and lower load is varied, for example, by Mallick et al. [26] or Santharam et al. [27]. Launay et al. considered the influence of temperature and humidity on their specimens’ performance [28].

### 1.3. Objectives and Novelty of This Contribution

While methods of so-called adaptive sampling are already successfully used in fields of simulation and can reduce the necessary number of passes, it is still rarely the case in experimental material data determination. One reason for this is certainly the existence of restrictions in the experimental world that are not found in simulation: for example, not all parameters can be changed at will.

This study describes an approach that uses artificial neural networks (ANNs) to characterise the fatigue behaviour of fibre-reinforced plastics more efficiently. The novelty of the approach lies in the fact that, by considering the model uncertainty, the areas of the parameter space are specifically fed with further experiments for which too few data are available. Restrictions are taken into account, such as the fact that it is not possible to produce an unlimited number of differently orientated samples.

## 2. Materials and Methods

All of the following observations were carried out on the short fibre-reinforced thermoplastic PBT GF30. This thermoplastic composite, based on polybutylene terephthalate, is reinforced with 30% glass fibres, which have fibre diameters of less than 10 µm and fibre lengths of up to 250 µm. To produce the sample material, plates were injection-moulded, from which the test specimens were then removed in various orientations by milling. The fibre orientation, depending strongly on the manufacturing process, was mostly parallel to the flow direction; details were calculated by means of an injection-moulding simulation [29,30].

### 2.1. Experiments and Evaluation Methods for Fatigue Life Prediction

#### 2.1.1. Experimental Assessment of Fatigue Life

The HCT 25 servo-hydraulic pulser from ZwickRoell, Ulm, Germany is utilised to characterise material behaviour under oscillating loads. It can apply axial tensile and compressive forces of up to 25 kN at a frequency of up to 30 Hz. In accordance with Bernasconi et al. [13,31], a stress ratio of 0.1 was applied to the minimum and maximum stress for the current characterisation, resulting in a pulsating tensile stress state. Loads were measured using piezoelectric load cells integrated into the testing machine.

All tests were conducted at a temperature of 23 °C. To prevent a significant rise in temperature due to internal friction, the test frequency was restricted to 4 Hz with a constant-load amplitude [32]. If the temperature increases by more than 10 °C, it can cause a rapid decrease in strength and unwanted thermal failure [12]. Therefore, an infrared thermometer was used to monitor and record the surface temperature of the specimen. If there is a critical increase in temperature, premature failure of the specimen is expected.

To prevent exactly this effect, the test frequency remained low. In addition, the sample surface was cooled with a constant flow of compressed air, which increased heat dissipation. In this way, there was no critical temperature increase in the samples—if an increase was detected, the test was declared invalid and discarded. The limit was drawn at a temperature increase of 5 °C.

**Figure 3:**
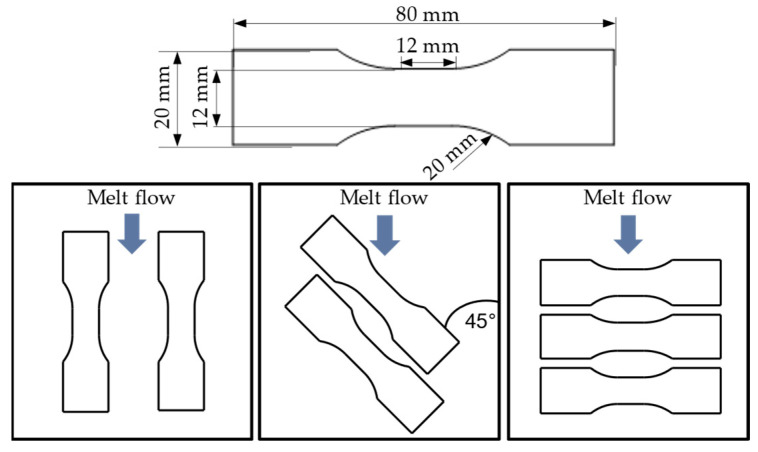
Specimen geometry and different locations within injection-moulded plates.

The tensile bar according to Becker [5] was used as the test specimen geometry, which was taken from injection-moulded plates with a thickness of 2 mm by milling. Figure 3 shows the exact geometry of the tension rods, along with their locations within the injection-moulded plates. The short fibres within the sheet were essentially oriented along the main direction of the melt flow. By taking the samples at different angles relative to this flow direction, it is possible to generate test specimens with different fibre orientations.

#### 2.1.2. Interpolation of Arbitrary Fibre Orientations in Fatigue Life

Our approach for interpolation was the use of a so-called S-N surface, which was derived and used by Witzgall and Wartzack in [33]. In this approach, using the results of different fibre orientations and including a failure criterion, a continuous surface was drawn that indicates the stress that can be carried. The fibre orientation angle was plotted here as the third dimension and the analytical function was derived using the Tsai–Hill criterion as follows:

The failure criterion according to Tsai–Hill [34], here, for transversal–isotropic material behaviour, is listed in Equation (1). It sets the existing fibre-parallel and -perpendicular stresses in the material in relation to the bearable values, $\sigma_{∥,max}$, $\sigma_{⊥,max}$ and $\tau_{∥⊥,max}$. If the limit value of 1 is exceeded, the material will fail.

(1) $$\frac{\sigma_{∥}^{2}}{\sigma_{∥,max}^{2}}+\frac{\sigma_{⊥}^{2}}{\sigma_{⊥,max}^{2}}-\frac{\sigma_{∥}\sigma_{⊥}}{\sigma_{∥,max}^{2}}+\frac{\tau_{∥⊥}^{2}}{\tau_{∥⊥,max}^{2}}=1$$

The rotation of the stress tensor results in the maximum stress that can be applied to a sample whose fibre orientation is rotated by an angle $\left(\theta \right)$ relative to the load direction:

(2) $$\sigma_{max}\left(\theta \right)={\left[\frac{\cos{\left(\theta \right)}^{2}\cdot \left(\cos{\left(\theta \right)}^{2}-\sin{\left(\theta \right)}^{2}\right)}{\sigma_{∥,max}^{2}}+\frac{\sin{\left(\theta \right)}^{4}}{\sigma_{⊥,max}^{2}}+\frac{\cos{\left(\theta \right)}^{2}\cdot \sin{\left(\theta \right)}^{2}}{\tau_{∥⊥,max}^{2}}\right]}^{-\frac{1}{2}}$$

The maximum stress in Equation (2) is therefore only dependent on the angle of orientation and the different material strengths. In addition, the basic equation of the S-N curve is as follows:

(3) $$\sigma_{f}\left(N\right)=\sigma_{f}\cdot N^{b}$$

The index *f* here denotes fatigue, *N* is the maximum number of cycles a specimen can withstand and the exponent *b* describes the slope of the S-N curve. Merging the equations provides a relationship that describes the fatigue strength of an arbitrarily orientated specimen as a function of the orientation angle and the number of cycles:

(4) $$\sigma_{f}\left(\theta ,N\right)={\left[\frac{\cos{\left(\theta \right)}^{2}\cdot \left(\cos{\left(\theta \right)}^{2}-\sin{\left(\theta \right)}^{2}\right)}{\sigma_{∥,f}^{2}}+\frac{\sin{\left(\theta \right)}^{4}}{\sigma_{⊥,f}^{2}}+\frac{\cos{\left(\theta \right)}^{2}\cdot \sin{\left(\theta \right)}^{2}}{\tau_{∥⊥,f}^{2}}\right]}^{-\frac{1}{2}}\cdot N^{b}$$

The strengths $\sigma_{i,f}$ listed in the denominator correspond in very good approximation to the respective static strengths, i.e., to a certain extent, the fatigue strength of a number of cycles of 1.

The approach described uses the assumption that the gradient of the S-N curves in the *N* direction, or the skew of the S-N surface, is constant regardless of the orientation angle. The good approximation of this assumption was proven for short-fibre-reinforced thermoplastics in [35]. In the case of a variable gradient over the fibre orientation, a dependence of the formerly constant exponent *b* towards an expression $b\left(\theta \right)$ was added to the approach in [36].

#### 2.1.3. Experimental Parameters and Their Restrictions

In order to clarify which parameters can generally be relevant in the present experiments, but also which restrictions they are subject to, they will be dealt with individually here.

Firstly, it is worth recalling the representation of the S-N curve, in which the number of cycles (*N*) is plotted on the abscissa, the horizontal axis, and the stress on the vertical axis, the ordinate—this is carried out for historical reasons, so to speak. It suggests that the number of cycles is the input parameter and the stress is the output parameter, as this is the usual arrangement. In reality, however, the choice of test load is the variable parameter and the tolerable number of cycles is the result of the experiment. The test load can be selected quite freely and continuously. Other parameters of the tests can be the stress ratio of the upper and lower load [26], the temperature [37] or the test frequency [31]. These can also essentially be regarded as continuous parameters.

In contrast, the next parameter to be used is the fibre orientation. As described above, the orientation is often only used in a few stages, for example, 0°, 45° and 90°. This is solely for organisational reasons: it must be noted that, as a rule, the samples must be taken from plates, a separate CNC program must be used for each different orientation, etc. In addition, the sample cuts must be ordered and it is simply not practicable to have each sample produced individually at any orientation angle. Accordingly, the fibre orientation, if it is to be modelled continuously, must be regarded as a quasi-discrete parameter that cannot be changed at will.

### 2.2. Method of Adaptive Sampling

Adaptive sampling is a method in which the design points still to be carried out are selected depending on the results already obtained. In other terms, this means that the test programme is not fixed from the outset, but is only determined gradually by looking at all the results [38]. According to the above definition, the pearl–string method, which was described earlier, is also basically an adaptive sampling method: here, the test force is changed depending on the previously endured number of cycles.

**Figure 4:**
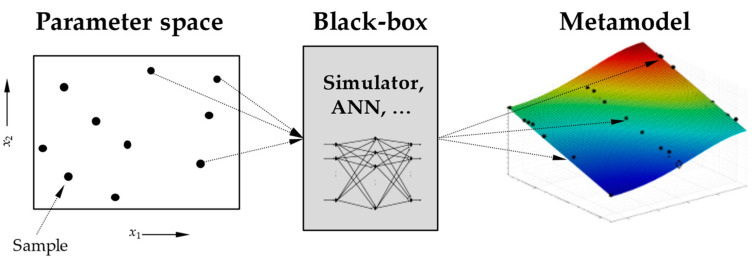
Modelling scheme: separate samples from a parametric domain are evaluated, then results are modelled continuously on the whole domain. Own work based on [39].

Regardless of the scientific discipline in question, a relation for the entire parameter space is created from individual samples in a parameter space by modelling, as shown in Figure 4. It is obvious that the accuracy of the determined model depends strongly on the samples. In addition, it is often very time-consuming to create the samples, either because the experimental effort is very high or because simulation times are very long. It is therefore a central goal to reduce the number of samples as much as possible, while generating a proficient surrogate model [39].

A prominent method that is widely used today in the field of computer-aided simulation is kriging, which was developed by Krige in connection with mining and geostatistics [40]. The “experiments” carried out there were on boring probes, which could only be made in limited quantities and therefore had to be placed in the best possible way in order to deliver the greatest possible gain in knowledge.

**Figure 5:**
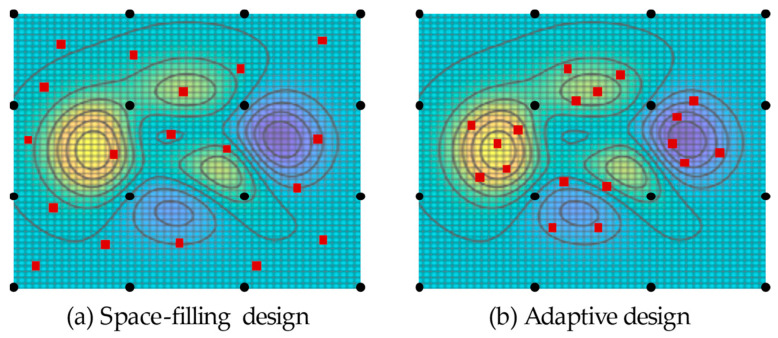
Comparison of space-filling design (**a**) and adaptive design (**b**). Initial samples are black dots; sequentially added samples are red squares. Own work based on [39].

In contrast to so-called space-filling designs, in which the samples are distributed evenly over the parameter space, the samples are concentrated at locations of interest during kriging, as Figure 5 shows.

The identification of these regions of interest, where additional experiments have to be carried out, is usually carried out by analysing the model uncertainty, for example, through covariances.

### 2.3. Artificial Neural Networks (ANNs)

Artificial neural networks (ANNs) have received a lot of attention in the last few decades due to their attractive capabilities in the modelling of complex nonlinear systems and decision making in addition to the advances of computing. The applications of neural networks are numerous and include many various fields, among which is engineering. ANNs have been used for the prediction of manufacturing systems’ performance [41], manufacturing process costs [42], photovoltaic solar integrated system efficiencies [43], space weather [44], outdoor sound transmission [45], stream flow [46] and wind waves [47].

In this study, we deal with modelling the fatigue experimental data of fibre-reinforced plastics using ANNs. The objective of the ANN model is to determine missing experimental information which would improve the accuracy of the model. Towards this end, the available experimental data are used to train an artificial neural network (ANN) model with one hidden layer, which gives a good approximation for continuous functions. Appropriate error analysis techniques can be applied to identify the regions of high errors, which initiate the need to have more experimental data in these regions.

Knowledge about the system dynamics and mapping characteristics is implicitly stored within the network that is trained with historical input–output process data. The input–output data for the fibre-reinforced plastics’ fatigue characteristics were collected experimentally. The simulation model approximates the real process well, except for a few input locations with higher error, which indicate the need for more experiments around these conditions. The ANN model is a nonlinear functional approximation of the real process [48]. Neural networks were originally inspired as being models of the human nervous system [49]. They have been shown to exhibit many abilities, such as learning, generalization and abstraction. These networks are used as models for processes that have input–output data available. Historical observations allow the neural network to be trained such that the error between the real output and the estimated (neural net) output is minimized. The model is then used for different purposes, among which are estimation, control and optimization.

**Figure 6:**
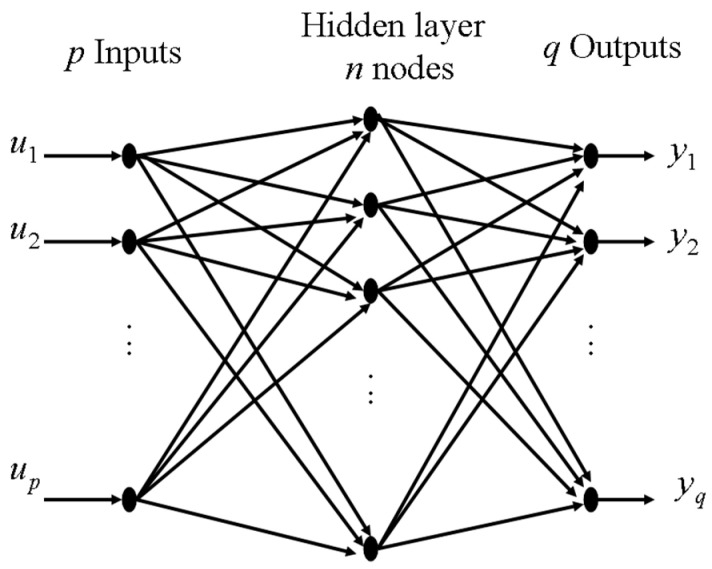
Neural network model representation.

The neural net structure is shown in Figure 6. The inputs feed forward through a hidden layer to the outputs [50]. The hidden layer contains processing units called nodes or neurons. Each neuron is described by a nonlinear sigmoid function. The inputs are linked to the hidden layer, which is, in turn, linked to the outputs. Each interconnection is associated with a multiplicative parameter called weight. The input weights are associated with the links between the inputs and the hidden layer, whereas the output weights are associated with the links between the hidden layer and the outputs.

In the case of available data, the stress and the orientation angle of the specimen are the setting variables of the test, i.e., the input parameters. The result of an experiment is the number of cycles until the specimen breaks. The influence of the input variable on the output variable is known from experience: with increasing load and with increasing deviation of the fibre orientation from 0°, the survival time of a sample will decrease. A general challenge for the use of ANNs in experimental contexts is the small number of available data points. For this reason, the nature of the ANN is limited to just a few hidden layers in order to avoid overfitting the data. This behaviour occurs when the number of hidden layers is greater than the complexity of the problem [51]. In this case, a number of hidden layers up to 5 is envisaged. The phenomenon of overfitting is also discussed, for example, by Almeida et al. [52] who, however, noticed it with a much higher number of hidden layers, more than 30. The ideal number of hidden layers for the problem at hand is determined in this study.

The Matlab software version 2022a was used to implement the ANN. The uncertainty of the model was evaluated using the least square error. Matlab initiates the weight matrix in each run and uses the backpropagation algorithm to find the optimum weights that will produce the best approximation of the real stress values as compared to the ANN output. This approach will provide the combinations of input parameters for which there is still the greatest uncertainty and for which it is suggested that further data be obtained by experimentation. Due to good practice with the method and, above all, the possible comparability of the error magnitude, standardised values are used for input and output variables.

However, the selection of further tests is subject to restrictions due to the method of sample preparation. This means that samples of any orientation cannot be produced one after the other. Instead, a production order must be initiated before the test series is carried out, from which the best outcome must be achieved. Requesting samples with any orientation is not feasible from this point of view. These restrictions must be taken into account by the ANN. In the case of the short fibre-reinforced samples made of PBT GF30, the orientations are 0°, 45° and 90°.

## 3. Results

In the following section, we want to compare the method of using an ANN in the test design with the traditional pearl–string method. This is undertaken on the basis of two data sets for the material PBT GF30, whose fatigue behaviour has been published in previous work [35].

**Figure 7:**
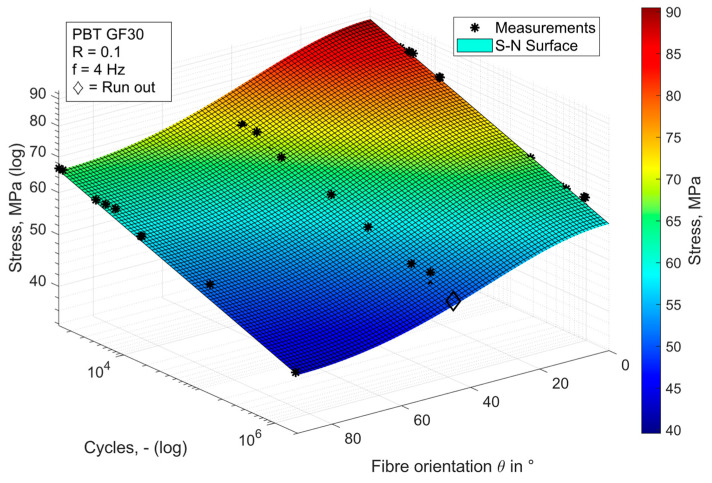
Representation of fatigue life prediction of PBT GF30 as S-N surface.

The available data were determined from a total of 34 test runs for the fibre orientations of 0°, 45° and 90°. The resulting S-N surface is shown in Figure 7. The following table also lists the parameters of the surfaces according to Equation (4). The results obtained are largely consistent with those found in the literature [21,53,54,55]. Oka et al. also found fatigue strengths between 100 MPa and 70 MPa for the range of 10^3^ to 10^6^ cycles in their tests on PBT GF30 [55]. A very similar slope of the S-N curves for all measured orientations was also found, for example, by Jain et al. [56,57].

For investigations with the neural network, numerous experiments with any combination of parameters would have to be carried out, which would not be possible in real experiments. In order to enable a large number of observations, further results are virtualised by calculation with noisy input data from the models already identified. Randomised values of the parameters within their confidence interval are used for this purpose. Equation (4) is used for implementation in Matlab, whereby the parameters $\sigma_{\|}$, $\sigma_{⊥}$, $\tau_{\|⊥}$ and b are used as random numbers in the scattering range (see Table 1).

Fatigue experimental data for the material PBT GF30 contains information about the stress, number of cycles and fibre orientation. The inputs are the stress and fibre orientation, whereas the output is the number of cycles endured. It is worthwhile to mention here that for the same values of stress and fibre orientation, experiments lead to varying values of the number of cycles, which follows a probability distribution that needs to be identified. Due to this fact, and with the use of neural networks to model the experimental data, it was observed that much more accurate models are obtained when the inputs are taken as the number of cycles and fibre orientation, with the output being the stress. From a computing point of view, the results should be acceptable as long as they can suggest the next experimental parameters to improve the predictive performance. Furthermore, the neural network can be reversed with the aid of optimization techniques to come back to the original input–output setup. The optimization procedure was attempted successfully in other various past occasions [58,59].

In total, 85% of the data patterns are used to train an artificial neural net model for the process, whereas the remaining 15% of data patterns are used to test the performance of the net. The data set was divided into a training region and test region as follows: the experimental data with the indices 7, 14, 21, … (every seventh set) were assigned to the test region. All other data were categorised in the training region. The ANN model was then updated and verified with newly generated data, which improved its accuracy considerably.

**Figure 8:**
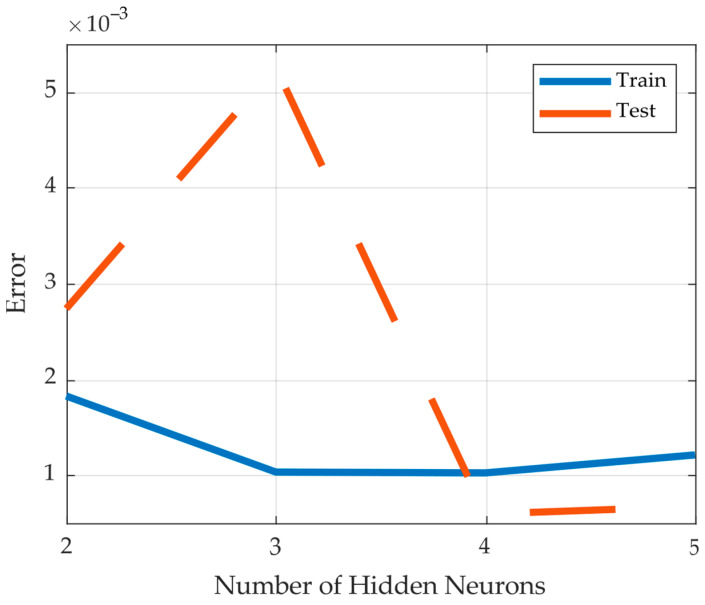
Square error as function of hidden neurons.

Training was carried out with the software package Matlab. The two inputs and the output were normalized to the range from 0 to 1 between the minimum and maximum values, respectively. The normalization procedure improves the accuracy of the neural net model. We ran experiments for different numbers of hidden neurons. It was observed that the quality of the results depends on the number of hidden neurons. The square error, *S*, is defined as

(5) $$S=\frac{1}{M}\overset{M}{\underset{1}{\sum }}{\left(y_{nn}-y_{r}\right)}^{2}$$

where *M* is the number of data points, *y_nn_* is the neural net output and *y_r_* is the real output. The square error for the training and test data is plotted as a function of the number of hidden neurons in Figure 8. Note that this function shows the optimum performance for the training and test region with four hidden neurons, where the normalized data training and test square errors were found as 1.02 × 10^−3^ and 0.60 × 10^−3^, respectively.

**Figure 9:**
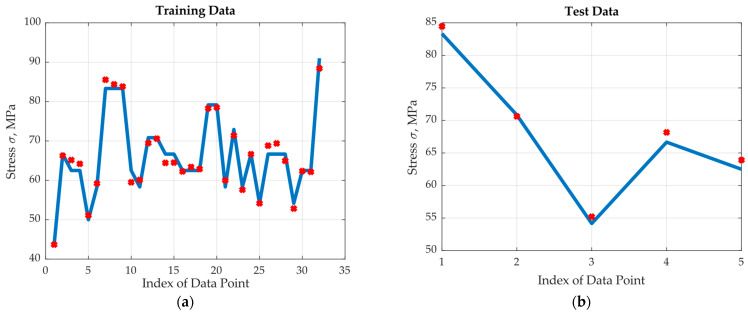
Results of the optimum ANN: (**a**) training data, (**b**) test data.

**Figure 10:**
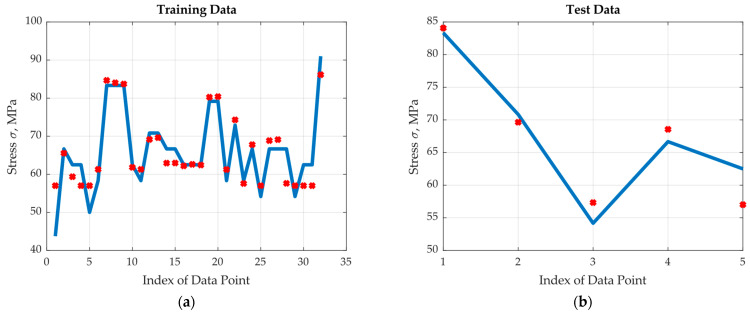
Results of the ANN with 1 hidden neuron: (**a**) training data, (**b**) test data.

The real output and the optimum (four hidden neurons) neural net approximated output are plotted in Figure 9a for the training region and in Figure 9b for the test region. Note that the two outputs are both very close to each other in the training region and close enough in the test region. To demonstrate the importance of the selection of the number of hidden neurons, the corresponding training and test modelling results for one hidden neuron are shown in Figure 10. The performance is much worse than the optimum one where the normalized data training and test square errors were recorded as 7.36 × 10^−3^ and 4.09 × 10^−3^, respectively.

**Figure 11:**
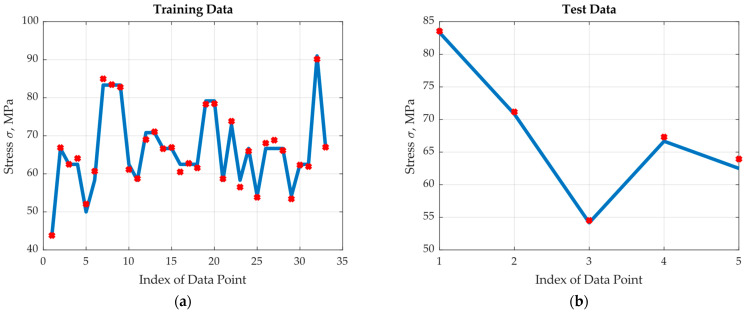
Results of the optimum ANN with additional experiments: (**a**) training data, (**b**) test data.

The results indicate the accuracy of the selected neural net. It was observed that the highest errors in the training region occur at a very low number of cycles, with more repetitions at the fibre orientation of 90°. This indicates the need for more experiments in these regions. Towards this end, one more set of experimental data including the two inputs and output was generated using a code that was calibrated to mimic the real experiment. The optimum neural network with four hidden neurons was retrained by adding the generated experimental data to the training region. The normalized data training and test square errors were reduced to about half values; namely, they were found to be 0.55 × 10^−3^ and 0.25 × 10^−3^, respectively. This substantial improvement is clear in Figure 11, where the real output and the optimum (four hidden neurons) neural net approximated output are plotted (a) for the training region and (b) for the test region. The followed methodology sets a new AI technique for suggesting parameters for the next experiments to improve the performance of the predicting model, which saves time and money and brings in more accuracy.

The squared errors for the training data and test data of the different configurations of the ANN are summarised in Table 2. The substantial improvement in the squared error, by a factor of seven, when comparing the ANN with one hidden layer with the ANN with four hidden layers, emphasises its suitability. The error was reduced from 7.36 × 10^−3^ and 4.09 × 10^−3^ for the training data and test data, respectively, to just 1.02 × 10^−3^ and 0.60 × 10^−3^. Adding another experiment at a point of high uncertainty, in the region of a 90° fibre orientation, could further improve the squared error, even approximately halving it. The error recorded then was only 0.55 × 10^−3^ and 0.25 × 10^−3^ for the training data and test data, respectively.

## 4. Discussion and Conclusions

The artificial neural networks (ANNs) showed promising results in proposing the parameters of the next fatigue experiments for the material PBT GF30 by observing high error regions. The goal was achieved successfully by switching the stress input with the number-of-cycles output, as it helped in improving the ANN’s accuracy substantially.

**Figure 12:**
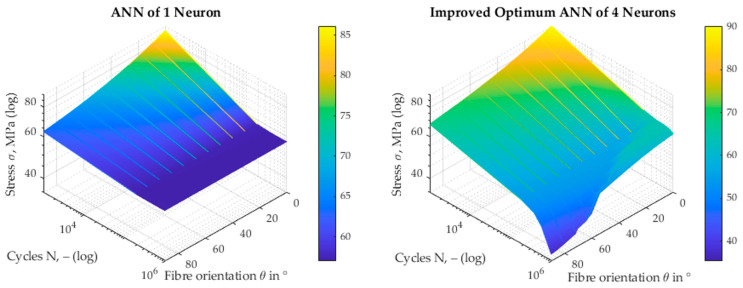
Comparison of resulting S-N surfaces from the 1-neuron ANN vs. optimum 4-neuron ANN.

This considerable improvement can also be recognised by looking at Figure 12, which shows the S-N surface in the $\left[N,\;\theta ,\;\sigma \right]$ space, on the left-hand side for the ANN with one hidden neuron and on the right-hand side for the optimum ANN with four neurons. It can be seen that, even with only one neuron, the ANN can map the dependence on the fibre orientation $\left(\theta \right)$ in the range of small cycles (*N*). However, for increasing numbers of cycles, it is noticeable that only a quasi-planar surface is displayed. The S-N surface, which was formed with the four-neuron ANN, provides a much better picture. Here, the dependence on both the fibre orientation (θ) and the number of cycles (*N*) appears to be mapped over a large range of cycles. The behaviour of the surface is striking in the range of high fibre orientations, θ→90°, and low stresses, σ. This is also the region of greatest uncertainty, where further experiments are necessary. The region comes to be identified by regionally high values of the squared error.

**Figure 13:**
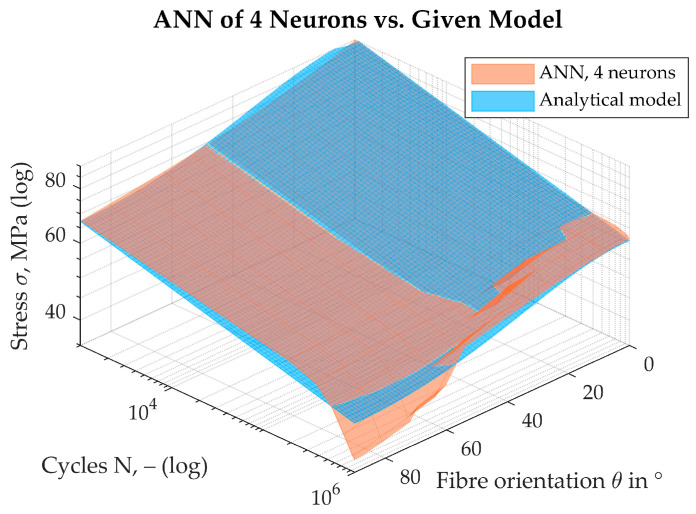
Comparison of resulting S-N surfaces from the optimum 4-neuron ANN vs. given model.

The quality of the ANN with four neurons is also confirmed when a direct comparison with the given model is considered, as can be seen in Figure 13. The S-N surface of the ANN, shown in a transparent orange colour, matches the given model in large areas of the parameter space. The points of low overlap are only found in the area that is also suggested for carrying out further experiments.

Optimization techniques can be applied to the ANN to return to the original input–output setup. The difference in performance when switching between input and output happened because the map is not one-to-one and the experimental data follow a probability distribution. Even though the results presented in this paper achieve the goal of experimental improvement, the neural network’s efficiency can be further improved by incorporating the probability distribution parameters within the inputs of the net. This study is worth undertaking in the future, as it will give more information on the fatigue life with success percentages. It has now been shown that the use of an ANN can make experimental design more efficient. After this proof of concept for just a few parameters, it will be possible to think further in the future: for example, influencing factors such as the temperature or stress ratio can also be taken into account. There seems to be much evidence to suggest that the use of ANNs to analyse uncertainties offers advantages. For the final modelling of the material behaviour in an S-N surface, it still seems reasonable to rely on the robust analytical model derived on the basis of material mechanics. Its stability, especially in the boundary regions of the parameter space, for example, due to fixed monotonicity and symmetry conditions, cannot be dismissed out of hand.

The main contribution to the novelty of this paper is the consideration of uncertainties in fatigue modelling with the help of the ANN in order to specifically underpin these areas with further experiments. It could be shown that the proposed approach works conceptually and can and should be taken up in the future.

For further research, it seems beneficial to combine the best of the worlds of neural networks, experimental data acquisition and analytical modelling. It will definitely be necessary in future work to validate the approach found with a detailed study of experimental investigations, possibly also with a material other than the one used here. Furthermore, it will be of interest in the future to extend the consideration of the ANN to other parameters, such as the temperature, the stress ratio (*R*) or the test frequency (*f*).

## Figures and Tables

**Table 1:** Parameter values of the fatigue model for PBT GF30.

Parameter	Value (Confidence Interval)
Fatigue strength, parallel direction, $\sigma_{\|}$	132.9 MPa (139.4 MPa, 126.3 MPa)
Fatigue strength, perpendicular direction, $\sigma_{⊥}$	102.3 MPa (107.0 MPa, 97.6 MPa)
Fatigue shear strength, $\tau_{\|⊥}$	67.5 MPa (71.1 MPa, 63.8 MPa)
Fatigue strength exponent, b	−0.057 (−0.053, −0.062)

**Table 2:** Summary of squared errors for the different configurations of ANN.

ANN with…	Squared Error for…	
	Training Data	Test Data
one hidden layer	7.36 × 10^−3^	4.09 × 10^−3^
four hidden layers	1.02 × 10^−3^	0.60 × 10^−3^
four hidden layers, additional experiments	0.55 × 10^−3^	0.25 × 10^−3^

## Data Availability

Data are contained within the article.
